# High Activation of γδ T Cells and the γδ2^pos^ T-Cell Subset Is Associated With the Onset of Tuberculosis-Associated Immune Reconstitution Inflammatory Syndrome, ANRS 12153 CAPRI NK

**DOI:** 10.3389/fimmu.2019.02018

**Published:** 2019-08-27

**Authors:** Polidy Pean, Janin Nouhin, Meng Ratana, Yoann Madec, Laurence Borand, Olivier Marcy, Didier Laureillard, Marcelo Fernandez, Françoise Barré-Sinoussi, Laurence Weiss, Daniel Scott-Algara

**Affiliations:** ^1^Immunology Unit, Institut Pasteur du Cambodge, Phnom Penh, Cambodia; ^2^Virology Unit, Institut Pasteur du Cambodge, Phnom Penh, Cambodia; ^3^Unité d'Épidémiologie des Maladies Émergentes, Institut Pasteur, Paris, France; ^4^Epidemiology and Public Health Unit, Institut Pasteur du Cambodge, Phnom Penh, Cambodia; ^5^Bordeaux Population Health, Centre Inserm U1219, Université de Bordeaux, Bordeaux, France; ^6^Department of Infectious and Tropical Diseases, University hospital, Nîmes, France; ^7^Médecin Sans Frontières, Geneva, Switzerland; ^8^Institut Pasteur, Paris, France; ^9^Hôpital Européen Georges Pompidou, Service d'Immunologie Clinique, Paris, France; ^10^Université Paris Descartes, Sorbonne Paris Cité, Paris, France; ^11^Unité Biologie cellulaire des Lymphocytes, Institut Pasteur, Paris, France

**Keywords:** HIV, tuberculosis, immune reconstitution inflammatory syndrome, gamma delta T cells, invariant NKT cells

## Abstract

**Background:** Human Immunodeficiency Virus 1 (HIV-1) and *Mycobacterium Tuberculosis* (Mtb) co-infected patients are commonly at risk of immune reconstitution inflammatory syndrome (IRIS) when initiating antiretroviral treatment (ART). Evidence indicates that innate immunity plays a role in TB-IRIS. Here, we evaluate the phenotype of Gamma-delta (γδ) T cells and invariant Natural Killer (iNK) T cells in tuberculosis-associated IRIS.

**Methods:** Forty-eight HIV+/TB+ patients (21 IRIS) and three control groups: HIV–/TB– (HD, *n* = 11), HIV+/TB– (*n* = 26), and HIV–/TB+ (*n* = 22) were studied. Samples were taken at ART initiation (week 2 of anti-tuberculosis treatment) and at the diagnosis of IRIS for HIV+/TB+; before ART for HIV+/TB-, and at week 2 of anti-tuberculosis treatment for HIV–/TB+ patients. γδ T cells and Invariant natural killer T (iNKT) cells were analyzed by flow cytometry.

**Results:** Before ART, IRIS, and non-IRIS patients showed a similar proportion of γδ^pos^ T and iNKT cells. HLA-DR on γδ^pos^ T cells and δ2^pos^γδ^pos^ T cells was significantly higher in TB-IRIS vs. non-IRIS patients and controls (*p* < 0.0001). NKG2D expression on γδ^pos^ T cells and the δ2^pos^γδ^pos^ T cell subset was lower in HIV+/TB+ patients than controls. CD158a expression on γδ^pos^ T cells was higher in TB-IRIS than non-IRIS (*p* = 0.02), HIV+/TB–, and HIV–/TB- patients.

**Conclusion:** The higher activation of γδ^pos^T cells and the γδ2^pos^γδ^pos^ T cell subset suggests that γδ T cells may play a role in the pathogenesis of TB-IRIS.

## Introduction

Tuberculosis (TB) and Human Immunodeficiency Virus (HIV) infection are serious global public health concerns. *Mycobacterium tuberculosis* (Mtb) primarily infects resident alveolar macrophages through various immune receptors (e.g., C-type lectin mannose receptors and scavenger receptors) expressed on the cell surface (1). In addition, DC-SIGN receptors also play a pivotal role in Mtb internalization by dendritic cells (DC) (2). By infecting antigen-presenting cells, such as macrophages and DC, Mtb can modulate antigen presentation, thereby affecting inflammation, DC cross-talk with other immune cells, and adaptive immune responses (3). Yet, knowledge of the interactions between Mtb and innate immune cells is limited. Increased access to antiretroviral therapy (ART) has significantly improved the clinical outcome of patients in resource-limited settings. However, between 4 and 54% of patients develop inflammatory responses, known as immune reconstitution inflammatory syndrome (IRIS), within the first few months of ART (4, 5). TB-associated IRIS (TB-IRIS) is thought to be directed toward Mtb antigens and is characterized by unexplained worsening or occurrence of symptoms or signs of TB post-ART initiation. Well-known risk factors associated with TB-IRIS include: low CD4^+^ T cell count below 200 cell/mm^3^ at the time of clinical diagnosis of co-infection (5, 6); short interval between onset of TB treatment and ART (5, 7); and, disseminated tuberculosis (5). However, there are no definite biomarkers to predict or diagnose this syndrome. It has been suggested that the pathogenesis of TB-IRIS involves both innate and adaptive immunity (4, 8), but the specific mechanisms of TB-IRIS pathogenesis remains unclear.

Patients with unmasking TB-IRIS display higher levels of Natural Killer (NK) cell activation and IL-8 than non-IRIS or Human Immunodeficiency Virus 1 (HIV-1)-monoinfected patients (9). Previously, we found that baseline capacity of NK cell degranulation was significantly higher in TB-IRIS patients vs. those without the syndrome, indicating a role of NK cells in the pathogenesis of TB-IRIS (10). Moreover, modification of the Gamma-delta (γδ) T cell repertoire, a well-known non-conventional T cell population that plays a role in the pathogenesis of Mtb infection, has also been reported in TB-IRIS patients (11). Gamma-delta T cells are innate-like T lymphocytes encompassing a small fraction (1–5%) of the circulating T lymphocyte pool. Unlike alpha-beta (αβ) T cells, γδ T cells express γ, and δ heterodimers of T cell receptors (TCR) associated with CD3 complexes and can recognize the lipid and glycolipid antigens produced by Mtb. Gamma-delta T cells also express various NK cell receptors (including NKG2D, killer immunoglobulin-like receptors KIRs) that play a role in the regulation of γδ T cell-mediated immune responses (12) including: cytolytic activity; pro- and anti-inflammatory cytokine production; and, the induction of a robust CD8^+^ T cell response via γδ T-APC crosstalk (13). The two major γδ T cell subsets are defined by their Vδ chains: Vδ1 and Vδ2. Most of the circulating γδ T cell pool is made up of the Vδ2^pos^γδ^pos^ subset (14). A higher proportion of γδ T cells and inversion of the Vδ1^pos^/Vδ2^pos^ ratio has been associated with chronic HIV infection (15).

Invariant natural killer T (iNKT) cells, which are CD1d-restricted glycolipid antigen reactive, can promote cell-mediated immunity against infection and tumors (16). Activation of iNKT cells results in rapid production of a large array of cytokines and chemokines which could be beneficial (16). Indeed, activation of CD1d-restricted iNKT cells protects against intracellular bacterial growth in Mtb infected mice (17); however, iNKT cell activity can also be harmful to the host in some diseases such as atherosclerosis and allergy (18). In HIV and TB mono-infections, iNKT cells are depleted and functionally impaired (19, 20), but partial reconstitution of iNKT cells during ART or anti-TB therapy has been observed (21). Interestingly, an elevated proportion of iNKT cell has been reported in TB-IRIS patients vs. non-IRIS control at the time of IRIS onset (22), but the exact role of iNKT cells in TB-IRIS is yet to be defined.

Therefore, we studied the peripheral levels, activation, the repertoire of γδ T cells and iNKT cells in TB-IRIS patients relative to that of non-IRIS patients in the CAMELIA clinical trial (23).

## Materials and Methods

### Patients and Samples

The study populations consisted of patients and control groups enrolled in a previously conducted NK cell study (CAPRI-NK/ANRS12153) (10), linked with the CAMELIA clinical trial (ANRS 1295-CIPRA KH001-DAIDS-ES ID 10425). The CAMELIA clinical trial was a prospective, randomized, multicenter, open-label, two-arm superiority trial conducted in Cambodia that demonstrated significant improvement in survival when ART was started 2 vs. 8 weeks following TB-therapy initiation. TB infection was diagnosed by a positive smear for acid-fast bacilli and was confirmed by culture for any clinical sample. TB-IRIS was defined as the unexplained worsening or re-occurrence of symptoms or signs of TB after ART initiation (e.g., fever, dyspnea, lymph-node involvement, or exacerbation of the diseases at other extra-pulmonary sites), as described elsewhere (5).

To avoid potential bias due to the different timing of ART initiation, 48 of the 128 HIV/TB co-infected patients who had received 2 weeks of TB therapy were randomly selected before ART initiation. Of the 48 HIV+/TB+ patients, 21 experienced TB-IRIS during the follow-up. Three control groups were also considered and served as the baseline for comparison: 11 adult healthy donors (HD) who were HIV serology negative and had no evidence of TB, 26 HIV-infected patients naïve of ART with no evidence of TB (HIV+/TB–) and 23 HIV serology negative TB positive patients (HIV–/TB+).

Gamma-delta T cells and associated subsets from HIV+/TB+ patients were assessed in cryo-preserved peripheral blood mononuclear cells (PBMCs) collected during the CAPRI-NK/ANRS12153 study at the time of ART initiation (week 2 of TB therapy), week 6 of ART (week 8 of TB therapy), and at IRIS diagnosis. Measurements were performed at the time of ART initiation for the HIV+/TB- group and at week 2 of TB therapy for the HIV–/TB+ group.

### Ethics Statements

This study was carried out in accordance with the recommendation of “French National Agency of Research on HIV/AIDS and Viral Hepatitis” Ethic Charter for Research version 2017 with written informed consent from all patients. All the patients gave written informed consent in accordance with the Declaration of Helsinki. The CAPRI-NK (ANRS 12153) study was approved by the “Cambodian National Ethics Committee for Health Research.” All participants gave their approval for the participation in the study by signing the dedicated informed consent form prior to any study procedure.

### Flow Cytometry Analysis

After thawing, PBMCs were stained with a combination of fluorochrome-labeled monoclonal antibodies for γδ T and iNKT cells. Expression of surface receptors was measured using a four-color FACSCalibur II flow cytometer (BD FACSCalibur Flow Cytometry System, RRID:SCR_000401).

Data acquisition was performed by BD CellQuest Pro (BD CellQuest Pro, RRID:SCR_014489) and analyzed using FlowJo Version 7.6.5 (FlowJo, Tree Star, Inc., RRID:SCR_000410).

Gamma-delta (γδ) and δ2^pos^γδ^pos^ T cells were defined as pan-γδTCR^pos^ and δ2^pos^γδ^pos^ by staining with anti-pan γδTCR-PC5 (Beckman Coulter Cat# IM2662, RRID:AB_131175) and anti-TCRVδ2-FITC (Beckman Coulter Cat# IM1464, RRID:AB_131019) monoclonal antibodies (mAbs). δ2^neg^ T cells, including γδ1^pos^ and γδ3^pos^ T cell subsets, was defined by gating δ2^neg^ population in the total γδ^pos^ T cell. Activation status and repertoire of γδ^pos^ and δ2^pos^γδ^pos^ T cells were determined by anti-HLADR-APC (BD Biosciences Cat# 559866, RRID:AB_398674), anti-CD158a, h-PE (KIR2DL1/DS1) (Beckman Coulter, Cat# A09778, RRID:AB_2801261), anti-CD158b1/b2, j-PE (KIR2DL2/L3) (Beckman Coulter Cat# IM2278U, RRID:AB_2728104), anti-NKG2D-PE (CD314) (Beckman Coulter, Cat# A08934, RRID:AB_2801262), and anti-NKG2C-APC (CD159c)(R and D Systems Cat# FAB138A, RRID:AB_416838) mAbs. Gating strategy for γδT cells is shown in Supplementary Figure 1.

Invariant natural killer T cells were defined using anti-CD3-FITC (Beckman Coulter Cat# A07746, RRID:AB_2801270) and anti-Vα24Jα18-APC (Thermo Fisher Scientific Cat# 17-5806-42, RRID:AB_10717252). iNKT cells subsets were identified using anti-CD56-PC5 (Beckman Coulter Cat# A07789, RRID:AB_1575976) mAbs, anti-CD4-FITC (BD Biosciences Cat# 555346, RRID:AB_395751), and anti-CD8-FITC (BD Biosciences Cat# 347313, RRID:AB_400279). In order to validate the sample acquisition for cytometry analysis, at least 100 events were recorded in the iNKT cell gate. Dead cells were gated out by forward and side scatter. Invariant natural killer T cell receptors were measured by staining with anti-NKp46-PE (CD335) (Beckman Coulter Cat# IM3711, RRID:AB_1575960), anti-CD161-PE (Beckman Coulter Cat# IM3450, RRID:AB_131250), anti-CD62L-PE (BD Biosciences Cat# 560966, RRID:AB_2033966), anti-CCR6-PE (BD Biosciences Cat# 559562, RRID:AB_397273), and anti-CD69-FITC (Beckman Coulter Cat#IM1943U; RRID:AB_2801272) mAbs.

The combination of fluorochrome-conjugated monoclonal antibodies used for immunostaining is shown as Supplementary Table 1. Representative dot plot of flow cytometry analysis of the marker expression on gamma delta T cells and invariant NKT cells is shown in Supplementary Figures 2E, 8F.

### Statistical Analyses

Statistical analysis was performed using GraphPad Prism software version 6.0e (GraphPad Software In., San Diego, CA, USA, RRID:SCR_002798). Phenotypic parameters obtained at baseline were first compared across the five groups by the Kruskal-Wallis test. If the test showed a significant difference, pair-wise comparisons were conducted. Phenotypic markers of IRS patients were compared to age, gender, CD4 T cells count and viral load matched non-IRIS patients using non-parametric (Mann Whitney U test). A *p*-value of <0.05 was considered statistically significant.

## Results

### Characteristics of Patients

At baseline, no significant differences were noted for sex (*p* = 0.83), age (*p* = 0.32), CD4 T-cell count (*p* = 0.86), and HIV-1 RNA viral load (*p* = 0.50) between TB-IRIS and non-IRIS patients (Table 1). Moreover, HIV/TB co-infected patients included in the present analysis had similar characteristics to those enrolled in the CAPRI-NK/ANRS12153 and CAMELIA clinical trials (10, 23). CD4 count was significantly lower in both TB-IRIS and non-IRIS HIV+/TB+ patients vs. HIV+/TB– patients (both *p* < 0.05). Although not significantly different, the HIV RNA plasma load in both TB-IRIS and non-IRIS patients tended to be higher than that of HIV+/TB– control patients.

**Table 1:** Baseline characteristics of patients.

	**HIV+/TB+ IRIS (*n* = 21)**	**HIV+/TB+ non-IRIS (*n* = 27)**	**HIV+/TB– (*n* = 24)**	**HIV–/TB+ (*n* = 22)**	**HIV–/TB– (*n* = 11)**
**Gender**					
Female *n* (%)	10 (47.6)	12 (44.4)	16 (61.5)	3 (13.6)	4 (36.3)
Male *n* (%)	11 (52.38)	15 (55.56)	10 (38.46)	19 (86.36)	7 (63.64)
**Age (years)**					
Median (IQR)	33 (28–38)	35 (29–43)	33 (30–41)	41 (32–56)	28 (25–38)
**CD4 (cells/mm**^**3**^**)**					
Median (IQR)	31 (17–51)	27 (18–108)	89 (20–167)*	–	–
**HIV-1 RNA VL (log10cp/mL)**					
Median (IQR)	5.7 (5.3–5.8)	5.6 (5.0–5.8)	5.0 (4.5–5.9)	–	–

IQR, Interquartile range.

* *p <0.05*.

### γδ^pos^ T Cells and δ2^pos^γδ^pos^ T Cell Subset in TB-IRIS

**Figure 1:**
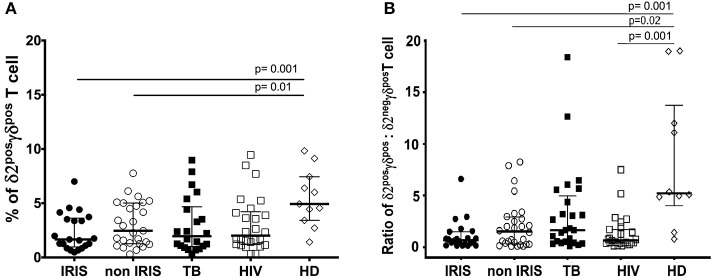
δ2^pos^γδ^pos^T cells and the ratio of δ2^pos^γδ^pos^: δ2^neg^γδ^pos^T cell in TB-IRIS, and non-IRIS at baseline. The proportion of δ2^pos^γδ^pos^ T cell **(A)**, the ratio of δ2^pos^γδ^pos^: δ2^neg^γδ^pos^ T cell **(B)** in TB-IRIS, non-IRIS, and control groups: [TB (TB+/HIV–), HIV (HIV+/TB–), HD (HIV–/TB–)] are shown. Results are expressed as median and 25–75% interquartile range. Significant *p*-values (*p* < 0.05) are indicated.

The proportion of γδ^pos^ T cells and δ2^pos^γδ^pos^ T cells was not different between TB-IRIS and non-IRIS patients at baseline (Figure 1A and Supplementary Figure 3A). However, both the level of δ2^pos^γδ ^pos^T cells and the ratio of δ2^pos^γδ^pos^: δ2^neg^γδ^pos^T cells were significantly lower in HIV and/or TB-infected patients vs. HD (Figures 1A,B). Moreover, δ2^pos^γδ^pos^: δ2^neg^γδ^pos^T cell ratio tended to be lower in TB-IRIS vs. non-IRIS patients [median (25–75% IQR): 0.69% (0.28–1.52) vs. 1.52% (0.31–2.91)] (*p* = 0.21), and HIV–/TB+ patients [median [(25–75% IQR): 1.65% (0.48–4.97)] (*p* = 0.26) (Figure 1B); however, the frequency of δ2^neg^γδ^pos^T cells in TB-IRIS and non-IRIS patients was not significantly different (Supplementary Figure 6A).

### Activation of γδ^pos^T Cells and δ2^pos^γδ^pos^ T Cell Subset in TB-IRIS Patients

Immune activation is a common feature of both HIV and TB infections, and elevated immune activation could play a role in the physiopathology of IRIS. Thus, we measured the level of HLA-DR as a marker of γδ T cell activation.

**Figure 2:**
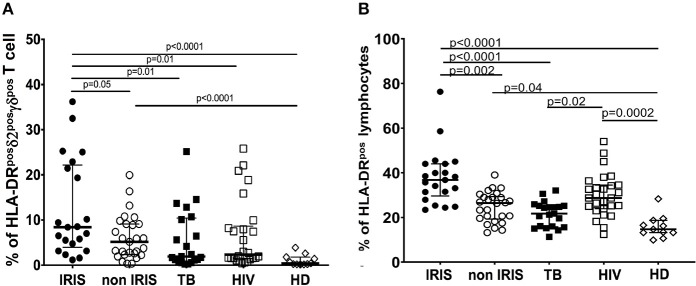
HLA-DR expression on δ2^pos^γδ^pos^ T cell, and total lymphocytes in TB-IRIS, and non-IRIS at baseline. The proportion of HLA-DR^pos^δ2^pos^γδ^pos^ T cell **(A)**, HLA-DR^pos^ on total lymphocytes **(B)** in TB-IRIS, non-IRIS, and control groups [TB (TB+/HIV–), HIV (HIV+/TB–), HD (HIV–/TB–)] are shown. The results are median and 25–75% interquartile range. Significant *p*-values (*p* < 0.05) are indicated.

At the time of ART initiation, HIV/TB co-infected patients showed significantly higher levels of HLA-DR on total lymphocytes, γδ^pos^T cells, and the δ2^pos^γδ^pos^T cell subset vs. HD (Figures 2A,B and Supplementary Figure 3D). Comparatively, TB-IRIS patients had significantly increased HLA-DR expression in δ2^pos^γδ^pos^T cell subset vs. those that were non-IRIS, HIV+/TB– or HIV–/TB+. There was no significant difference in HLA-DR expression on δ2^neg^γδ^pos^T cells between TB-IRIS and non-IRIS patients (Supplementary Figure 6B). However, levels of HLA-DR were significantly higher [median (25–75% IQR): 13.5 (7.5–26.0)] in TB-IRIS patients than HIV–/TB+ patients (*p* = 0.004) and HD (*p* = 0.04) and tended to be higher than in non-IRIS [median (25–75% IQR): 9.8 (6.3–17.4)] and HIV+/TB– patients (Supplementary Figure 6B).

We next examined the evolution of γδ^pos^ T cell activation from the time of ART initiation to the IRIS event. Samples from eight TB-IRIS patients and six non-IRIS controls were available for longitudinal analysis. Expression of HLA-DR on γδ^pos^ T cells significantly decreased from baseline to the onset of IRIS in TB-IRIS patients (*p* = 0.02), whereas it remained unchanged in non-IRIS patients (Supplementary Figure 4A). In addition, the delta value of HLA-DR on γδ^pos^ T cells from baseline to IRIS onset tended to be lower than those observed in non-IRIS patients (*p* = 0.06) (Supplementary Figure 4B).

### NKG2D and NKG2C Expression on γδ^pos^ T Cells in HIV/TB Co-infected Patients

**Figure 3:**
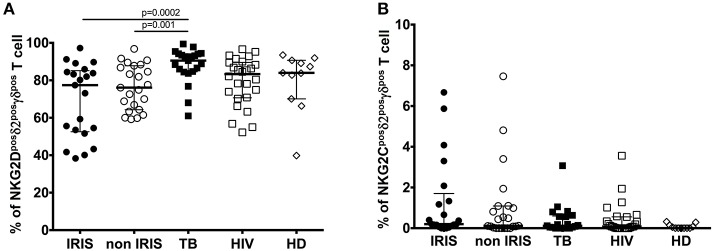
NKG2D, and NKG2C expression on δ2^pos^γδ^pos^ T cell in TB-IRIS and non-IRIS at baseline. The proportion of NKG2D^pos^δ2^pos^γδ^pos^ T cell **(A)**, NKG2C^pos^δ2^pos^γδ^pos^ T cell **(B)** in TB-IRIS, non-IRIS, and control groups [TB (TB+/HIV–), HIV (HIV+/TB–), and HD (HIV–/TB–)] are shown. The results are median and 25–75% interquartile range. Significant *p*-values (*p* < 0.05) are denoted.

Gamma-delta T cells share many features with NK cells, including the expression of activating and inhibitory NK-cell receptors as well as cytotoxic functionality. Therefore, we assessed NKG2D and NKG2C expression on γδ^pos^ T cells during TB-IRIS (Figures 3A,B and Supplementary Figures 3E, 5, 6C).

NKG2D is a C-type lectin-like NK receptor that recognizes cellular stress protein-ligand MICA-B and ULBP. Expression of NKG2D receptors on γδ T cells play a role in co-stimulating TCR-mediated activation, resulting in pro-inflammatory cytokine production and cytotoxic activity (24). Representative flow plots are shown in Supplementary Figures 2C,D. Although NKG2D expression on total γδ^pos^ (Supplementary Figure 3E), δ2^pos^γδ^pos^ (Figure 3A) and δ2^neg^ γδ^pos^ T cells (Supplementary Figure 6C) was not significantly different between IRIS and non-IRIS patients at baseline, NKG2D levels of IRIS patients were lower than those in the control groups, and IRIS patients exhibited lower median values vs. non-IRIS patients. At IRIS onset, NKG2D expression on the surface of γδ^pos^ T cells and δ2^pos^γδ^pos^ T cells was not significantly different between IRIS and non-IRIS control (Supplementary Figures 5A,B). The observed decrease in expression of NKG2D on γδ T cells in HIV and/or TB infection has been previously described (10, 25, 26).

NKG2C is the killer cell lectin-like receptor C2 (KLRC2) specific for HLA-E. Changes in expression of NKG2C has been reported in HIV-infected and TB patients (27, 28). NKG2C expression on γδ^pos^ T cells and the δ2^pos^γδ^pos^ T cell subset was low in all patient groups as well as in HD (Figure 3B and Supplementary Figure 3F). However, it was higher for the patients whatever infection vs. HD. Levels of NKG2C on δ2^neg^γδ^pos^ T cells were similar for all groups (Supplementary Figure 6D).

#### Killer Cell Immunoglobulin-Like Receptors (KIRs): CD158a and CD158b on γδ T Cells in TB-IRIS

KIRs regulate γδ T cell activation and function (29). Moreover, changes in KIR gene expression are related to the evolution of HIV infection (30–32). KIR expression on γδ T cells was reported to be reduced in IRIS vs. non-IRIS patients in a European cohort (11). Therefore, we measured KIR expression on γδ T cells in our patient cohort.

**Figure 4:**
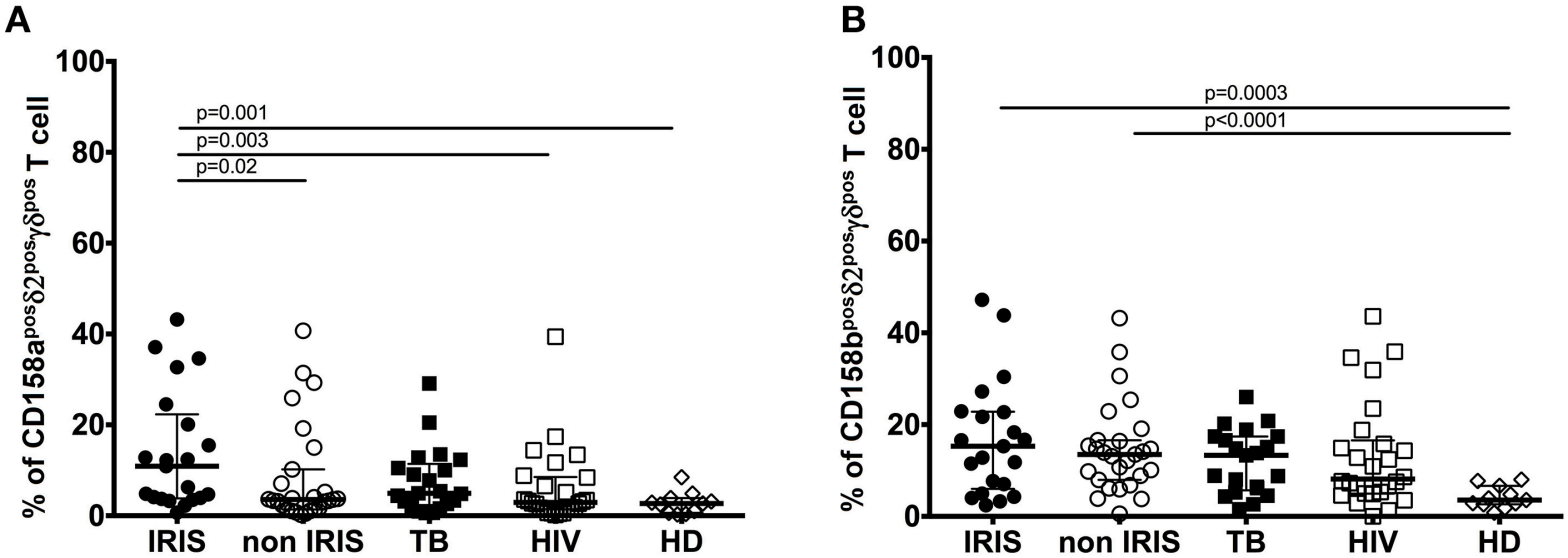
Killer Immunoglobulin-like receptors CD158a, and CD158b expression on δ2^pos^γδ^pos^ T cell subset in TB-IRIS and non-IRIS at baseline. The proportion of CD158a^pos^δ2^pos^γδ^pos^ T cell **(A)**, CD158b^pos^δ2^pos^γδ^pos^ T cell **(B)** in TB-IRIS, non-IRIS, and control groups [TB (TB+/HIV–), HIV (HIV+/TB–), and HD (HIV–/TB–)] are shown. The results are median and 25–75% interquartile range. Significant *p*-values (*p* < 0.05) are denoted.

At baseline, expression of CD158a on total γδ^pos^ T cells (Supplementary Figure 3B) close to be significant in TB-IRIS patients vs. non-IRIS[median (25–75% IQR): 12.40 (7.78–19.60) vs. 6.30 (2.94–14.50), *p* = 0.05]; however, CD158a expression on γδ^pos^ T cells was significantly higher for TB-IRIS patients than HD [median (25–75% IQR): 4.45 (1.68–8.30)] (*p* = 0.001). Further, CD158a expression on δ2^pos^ γδ^pos^ T cells was significantly higher in TB-IRIS patients [median (25–75% IQR): 10.90 (3.82–22.30)] than in non-IRIS patients [(median (25–75% IQR): 3.56 (1.38–10.20)] (*p* = 0.02), HIV+/TB– patients (*p* = 0.003), and HD (*p* = 0.001) (Figure 4A). There was no difference in CD158b expression on γδ^pos^ T cells or the δ2^pos^γδ^pos^ T cell subset between TB-IRIS and non-IRIS patients (Figure 4B and Supplementary Figure 3C). Nevertheless, CD158b expression on γδ^pos^ and δ2^pos^γδ^pos^ T cells tended to be higher for all infected patients vs. HD. Representative flow plots of CD158a and CD158b expression on γδ^pos^ T cells and the δ2 subsets are shown in Supplementary Figures 2A,B.

### Invariant NK T Cells in HIV/TB Co-infected Patients

Several different approaches have been used to identify invariant Natural killer T cells (iNKT) in the literature (18); however, the anti-Vα24Jα18 CDR3 loop TCR monoclonal antibody (6B11) has been suggested to be highly specific for the identification of human iNKT cells (33). In addition, a portion of CD3^+^iNKT cell express CD56, and this cell subset has been reported to be cytotoxic and produce regulatory cytokines (34).

iNKT cells and iNKT cell subsets were defined by the co-expression of CD3 and Vα24Jα18. The gating strategies for analysis of whole and CD56^+^iNKT cell subset are shown Supplementary Figure 7.

**Figure 5:**
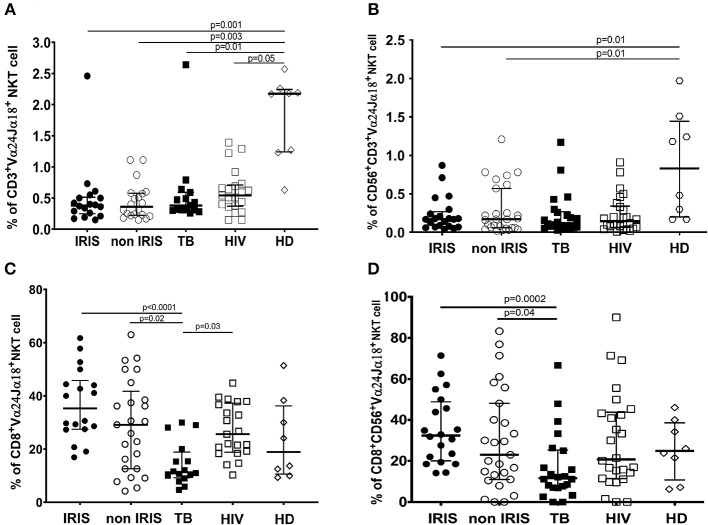
The phenotype of circulating invariant NKT cells and CD56^+^NKT cell subset in TB-IRIS and non-IRIS at baseline. The proportion of circulating Vα24Jα18^+^ iNKT cells **(A)**, or CD56^+^Vα24Jα18^+^ iNKT cells among total CD3^+^ T cells **(B)**; and proportion of CD8^+^ iNKT cells among total Vα24Jα18^+^ iNKT cells **(C)** or CD8^+^CD56^+^iNKT cells among Vα24Jα18^+^CD56^+^ **(D)**, in TB-IRIS, non-IRIS, and control groups [TB (TB+/HIV–), HIV (HIV+/TB–), and HD (HIV–/TB–)] are shown. The results are shown as median and 25–75% interquartile range. Significant *p*-values (*p* < 0.05) are indicated.

Number of iNKT cells and CD56^+^iNKT cells were lower in all groups of infected patients vs. HD (*p* ≤ 0.05) (Figures 5A,B). Both total iNKT cells and CD56^+^iNKT cells were similar at baseline between TB-IRIS and non-IRIS patients.

Although both CD8^+^iNKT cell and CD8^+^CD56^+^NKT cell subset levels were not a difference between TB-IRIS and non-IRIS, they were decreased in Mtb infected patients and were significantly different between IRIS (Figures 5C,D). Also, CD4^+^iNKT cells at baseline were not the different between TB-IRIS and non-IRIS. However, CD4^+^iNKT cells and the ratio of CD4^+^iNKT: CD8^+^iNKT was lower in HIV and/or TB infection than HIV–/TB+ and HD (Supplementary Figures 9A,B).

**Figure 6:**
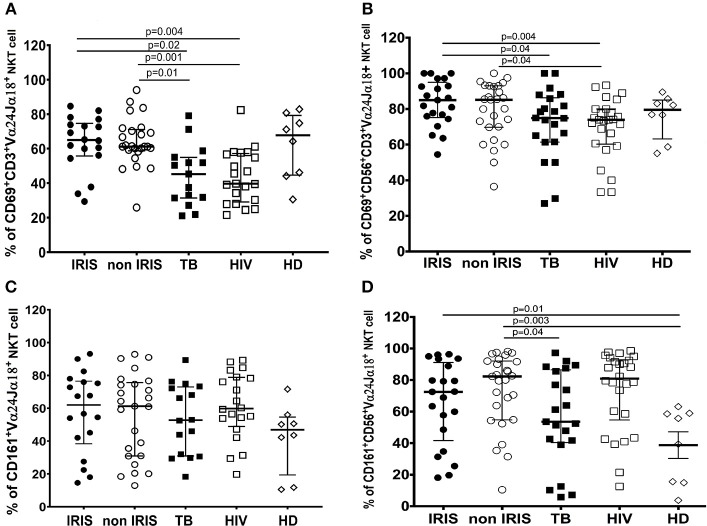
CD69, and CD161 expression on iNKT cells and CD56^+^iNKT cell subset in TB-IRIS and non-IRIS at baseline. The proportion of CD69 expression on CD3^+^Vα24Jα18^+^ iNKT cells **(A)**, and on CD56^+^CD3^+^Vα24Jα18^+^ **(B)**; the proportion of CD161 expression on Vα24Jα18^+^ iNKT cells **(C)**, and on CD56^+^CD3^+^Vα24Jα18^+^ iNKT cells **(D)** in TB-IRIS, non-IRIS, and control groups [TB (HIV–/TB+), HIV (HIV+/TB–), and HD (HIV–/TB–)] are shown. The results are median and 25–75% interquartile range. Significant *p*-values (*p* < 0.05) are presented.

We also studied the activation of iNKT cells and CD56^+^iNKT cells by measuring the expression of several markers. CD69 levels at baseline were similar between TB-IRIS and non-IRIS patients, HIV/TB co-infected patients had increased number of both activated iNKT cells and CD56^+^iNKT cells compared to mono-infected TB or HIV patients (Figures 6A,B). Further, expression of CD161 on iNKT cell and CD56^+^iNKT cells did not differ between TB-IRIS and non-IRIS patients. However, CD161 expression on both iNKT cells and CD56^+^iNKT cells was higher in mono-infected HIV or HIV/TB patients vs. HD (Figures 6C,D). No significant differences in the expression of NKp46, CCR6, CD62L on CD56^+^iNKT cells subset were observed. Representative flow plots are depicted in Supplementary Figure 8.

## Discussion

Diagnosis of TB-IRIS is currently based purely on clinical findings. Thus, a better understanding of the physiopathology and risk of TB-IRIS in HIV/TB co-infected patients is vital for the development of prognostic or predictive tests. While the restoration of immunity, particularly T cell immunity, against Mtb is often cited as playing a central role in TB-IRIS, evidence is contradictory and other immune cells may play a vital role in syndromic development. Several lines of evidence indicate that innate immunity plays a crucial role in TB-IRIS. For example, abundant infiltration of CD68^+^ macrophages was observed in the post-mortem lung tissue from a confirmed TB-IRIS case (35). In addition, a high level of caspase-1 expression by CD64^+^ monocytes and elevated plasma IL-1β and IL-18 levels have been reported (36), along with elevated levels of IL-6 and C-reactive protein (8, 37, 38). TLR and TREM-1-induced inflammasome signaling have also been reported to be up-regulated in TB-IRIS patients (8). In addition to monocytes/macrophages, NK cells and invariant natural killer T cells may also contribute to the pathogenesis of TB-IRIS (9, 10, 22, 36). Patients with unmasking TB-IRIS have increase NK cell activation and IL-8 expression vs. non-IRIS or HIV-1-mono-infected controls. Our previous results show a large increase in NK cell activity associated with TB-IRIS, further bringing to light the role of innate immunity in IRIS (10). Others have subsequently confirmed these results (9, 22, 36). Further, modification of the γδ T cell repertoire (11) and an elevated proportion of iNKT cell have been reported in TB-IRIS patients (11, 22). Therefore, we extended our NK cell study to γδ T cells and iNKT cells.

In our study, the proportion of peripheral γδ^pos^T cells did not differ at baseline between TB-IRIS and non-IRIS patients; however, there was a significantly higher expression of HLA-DR on γδ^pos^T cells and δ2^pos^γδ^pos^T cells at baseline in TB-IRIS patients. Consistent with previous reports, our results demonstrate that TB-IRIS patients have highly activated γδ^pos^T cells and δ2^pos^γδ^pos^T cells at baseline, and that immune activation is higher in HIV/TB co-infection vs. TB or HIV mono-infection alone (39). Moreover, we show an increase in CD158a (KIR2DL1/DS1) expression on γδ^pos^T cells in TB-IRIS patients. We did not find any difference between iNKT cells and CD56^+^iNKT cells in TB-IRIS and non-IRIS patients at baseline. Due to the limitations of the retrospective nature of our study and precious sample sizes, we, unfortunately, cannot extend our conclusions. Further work will be conducted to include a greater number of markers and the function of the different cell types. Moreover, the evolution of these markers will be followed in detail at more time points. The evolution of HLA-DR on γδ^pos^ T cells from baseline to IRIS time (week 8 of ART) significantly declined in IRIS patients. Although the levels of HLA-DR on γδ^pos^ T cells were lower in non-IRIS patients at baseline, we also observed a non-significant decrease of activated γδ^pos^ T cells. However, the small number of samples tested might be limited to see a significant difference. This decrease could be explained by the effect of ART in both groups of patients. However, we cannot exclude the role of regulatory populations in the IRIS group.

As previously described (40–42), we also observed decreased proportions of δ2^pos^γδ^pos^T cells, decreased expression of NKG2D on γδ^pos^T cells, and depletion of both iNKT cells and CD56^+^iNKT cells in TB and/or HIV infected patients vs. HD, regardless of the infection condition.

Gamma-delta T cell activation is a known feature of active tuberculosis and is also observed in HIV/TB co-infected patients (11, 42). In a European setting, Bougarit et al. reported higher levels of δ2^pos^γδ^pos^T cells in TB-IRIS patients. Furthermore, they found that this subset exhibited strong IFN-γ production. They thus postulated that δ2^pos^γδ^pos^T cell populations could be involved in TB-IRIS via the secretion of pro-inflammatory cytokines (11). Here, we observed a trend toward lower levels of δ2^pos^γδ^pos^T cells in TB-IRIS subjects, although it was not significant. It has been shown that interactions between γδ^pos^T cells, NK cells, DCs can control immune activation and inflammation. δ2^pos^γδ^pos^T cells play an important role in human immunity to *M. tuberculosis* and HIV-1. Defects in one type of these cell types can lead to an imbalance in the cross-talk between γδ T cells, NK cells, and DCs and consequently a loss of control in immune activation and inflammation (43–45). Our data showed significantly increased levels of γδ^pos^T cell and δ2^pos^γδ^pos^T cell activation at baseline. Mtb antigens could be partly responsible for this activation. Moreover, δ2^pos^γδ^pos^T cells dysfunction has been described in tuberculosis and HIV-1 infection (42). However, this result differs from the findings of Bourgarit et al. who did not find any difference between TB-IRIS and non-IRIS patients in terms of HLA-DR expression on γδ T cells in a French population. This conflicting result could be explained by the fact that basal innate immune activation in HIV/TB co-infected Cambodian patients is higher than that observed in European patients (11). Moreover, the background stimulation of innate immunity by local endemic pathogens could also play a role. Future studies will test the functional capacity of γδ T cells from Cambodian patients for comparison.

Modulation of NKG2D expression in TB and HIV/TB co-infected patients has previously been described (26, 46), and correlates with a loss of NK cell function (10, 47). Decreased expression of NKG2D on δ2^pos^γδ^pos^T cell at baseline in TB/HIV infected patients could be explained by the increased level of NKG2D ligands on TB infected macrophages (48), leading to the internalization of NKG2D receptors (25, 49, 50). However, a limitation of our study was the absence of soluble NKG2D ligand measurements (50). Nevertheless, we did not observe the lower expression of NKG2D on δ2^pos^ γδ^pos^T cells at the time of TB-IRIS diagnosis, suggesting the restoration of γδ T-cell functions (51, 52).

Perhaps, the most intriguing result from this study is of the increased CD158a (KIR2DL1/DS1) expression on δ2^pos^γδ^pos^ T cells in patients who develop TB-IRIS. CD158a has both inhibitory and activating properties. Expression of this KIR correlates with better control of HIV replication (31, 53). Interestingly, our data contrast with those obtained in another study that reports a higher baseline proportion of δ2^pos^γδ^pos^ T cells lacking KIR expression in TB-IRIS patients (11). However, we cannot distinguish between activating or inhibitory KIRs, making the interpretation of this result difficult. As modulation of KIR expression has been described during HIV infection (30–32), the increase in CD158a expression in TB-IRIS patients could be related to better preservation of γδ T cells, as we already showed for NK cells (10). A detailed study of γδ T cell populations will be necessary to better define their role in TB-IRIS.

Invariant NKT and CD3^+^CD56^+^NKT cells subset have also been previously described in HIV and/or TB infected patients (34, 54, 55). One study suggests depletion iNKT cells occurs early in HIV infection, and that this loss appears to be persistent (56). In contrast to HIV infection alone, one report suggests an increase in iNKT cell levels associated with increased plasma levels of granzyme B and perforin in IRIS patients (22, 57). This increase could be due to a rapid recovery of iNKT cells by ART (58); however, we did not observe any difference between TB-IRIS and non-IRIS in terms of the proportion of iNKT cells and CD56^+^iNKT cells, their level of maturation, or activation at baseline of TB-IRIS onset. These results suggest that there is no association between iNKT cells and TB-IRIS development in the context of our cohort.

The interaction of the CD161 receptor with its ligand, lectin-like transcript−1 (LLT-1), has been reported to have both inhibitory and co-stimulatory effects (59) and has been associated with IFN-γ and IL-17 secretion by CD4^+^ T cells (60). We observed lower levels of iNKT cells, regardless of the infection (HIV or TB), vs. HD. This finding was not surprising, as depletion of peripheral-blood iNKT cells has been reported in several studies (21, 61). However, the mechanisms of such depletion are not well-understood. A study conducted in HIV-infected patients proposed that depletion of iNKT cells could be related to the apoptosis induced by the Fas/FasL pathway (62). In the context of Mtb infection, the decrease of peripheral iNKT cells could be due to their migration to the lung, where they contribute to granuloma formation (21). In addition, we observed increased expression of CD161 and CD69 on iNKT cells of HIV/TB co-infected patients. These results are consistent with those of Snyder-Cappione et al., who found that the frequency of NKT cells expressing CD161 negatively correlated with the production of both TNF-α and IFN-γ (61). Also, it has been demonstrated that innate CD4^+^Vα24^+^ NKT cells regulated by IL-7 typically produce Th1- and Th2-associated cytokines (63). The depletion and functional impairment of CD4^+^iNKT cells and CD8^+^ iNKT cells during HIV infection have been described in several studies (21). We did not measure iNKT cell activity but it is possible that the cytokine-producing capacity of iNKT cells could be impaired during chronic HIV/TB co-infection and related to the decrease of total iNKT cells observed in infected patients.

Our results provide further evidence for the involvement of innate immunity in the pathogenesis of IRIS in HIV/TB co-infected patients. More studies on the role of innate immunity could be beneficial in the search for biomarkers of TB-IRIS.

## Data Availability

All datasets generated and analyzed in this study are included in the manuscript and Supplementary Files.

## Ethics Statement

This study was approved by the Cambodian National Ethics Committee for Health Research and informed consent was obtained from all participants.

## Author's Note

The results of the present study have been presented (Abstract ID 261) at the International Symposium of The Pasteur Institutes International Network, October 2015, Paris, France.

## Author Contributions

FB-S, LW, PP, and DS-A conceptualized and designed the study. OM, DL, LW, FB-S, and DS-A contributed to the experimental design and provided intellectual input. MR, JN, and PP performed experiments and data collection. MF, DL, OM, and LB were clinical investigators of the CAMELIA clinical trial. YM analyzed data and performed statistical analyses. JN, YM, LW, PP, and DS-A wrote the manuscript. LB was in charge of ethical issues. All authors revised the manuscript.

### Conflict of Interest Statement

The authors declare that the research was conducted in the absence of any commercial or financial relationships that could be construed as a potential conflict of interest.
