# Supplementary material for: High Activation of γδ T Cells and the γδ2pos T-Cell Subset Is Associated With the Onset of Tuberculosis-Associated Immune Reconstitution Inflammatory Syndrome, ANRS 12153 CAPRI NK
Source: Front Immunol. 2019 Aug 27;10:2018. doi: 10.3389/fimmu.2019.02018 (PMC6718564; doi:10.3389/fimmu.2019.02018)
Supplement: Supplementary file 1 [file Data_Sheet_1.docx]

Supplementary Material


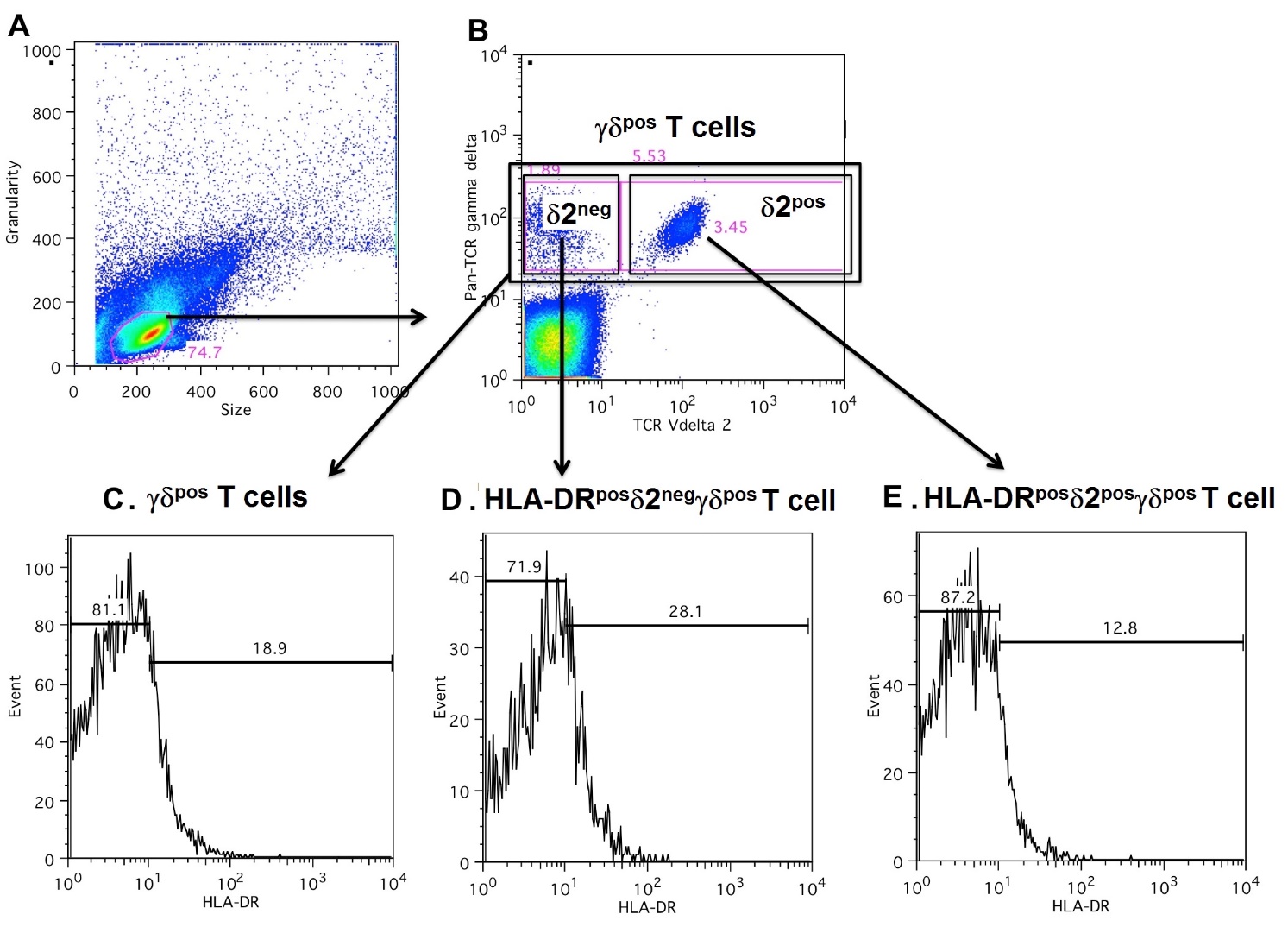


**Supplementary Figure 1 (Figure. S1).** **Gating strategy of γδ^pos^ T cells in peripheral blood mononuclear cells.** γδ^pos^ T cells are analyzed in the lymphocyte gate (granularity versus size) **(A.)**. δ2^pos^ and δ2^neg^ T subpopulations are gated in γδ^pos^ T cells **(B.)**. The markers (e.g HLA-DR) of each population displayed in the histograms **(C.)**; **(D.)** and **(E.)** respectively.


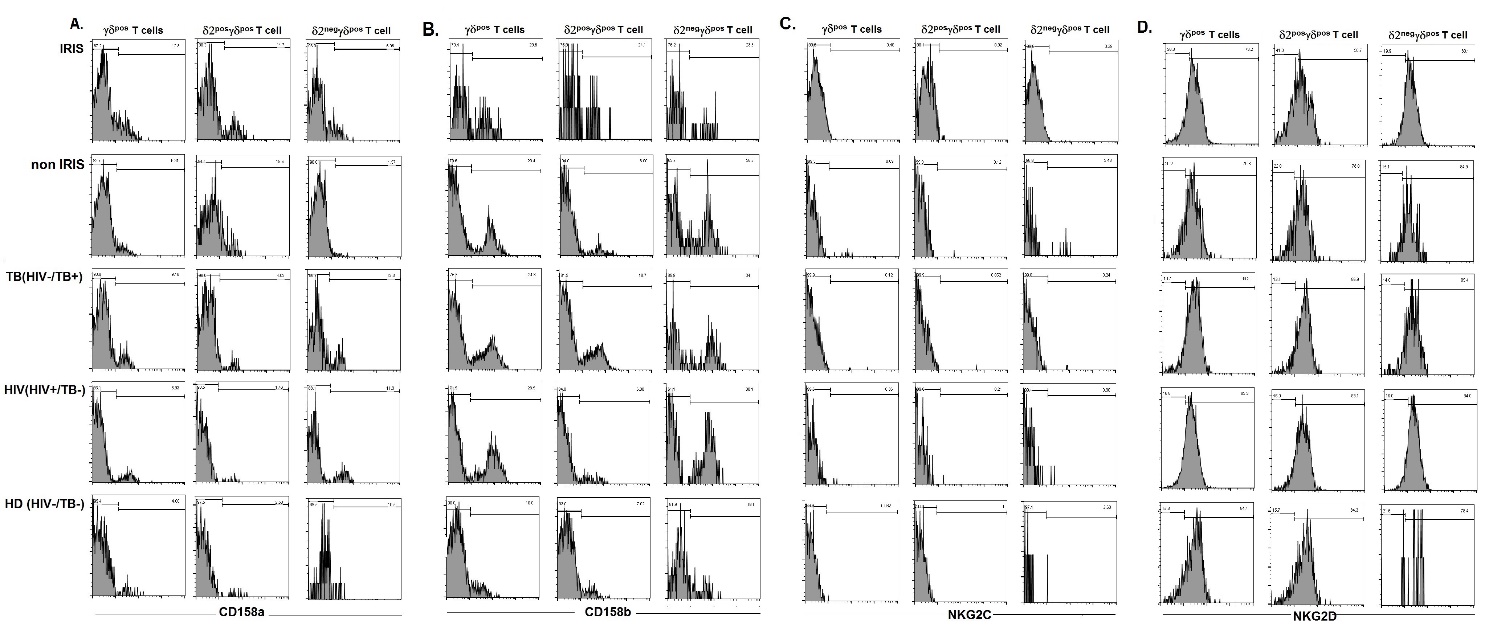


**
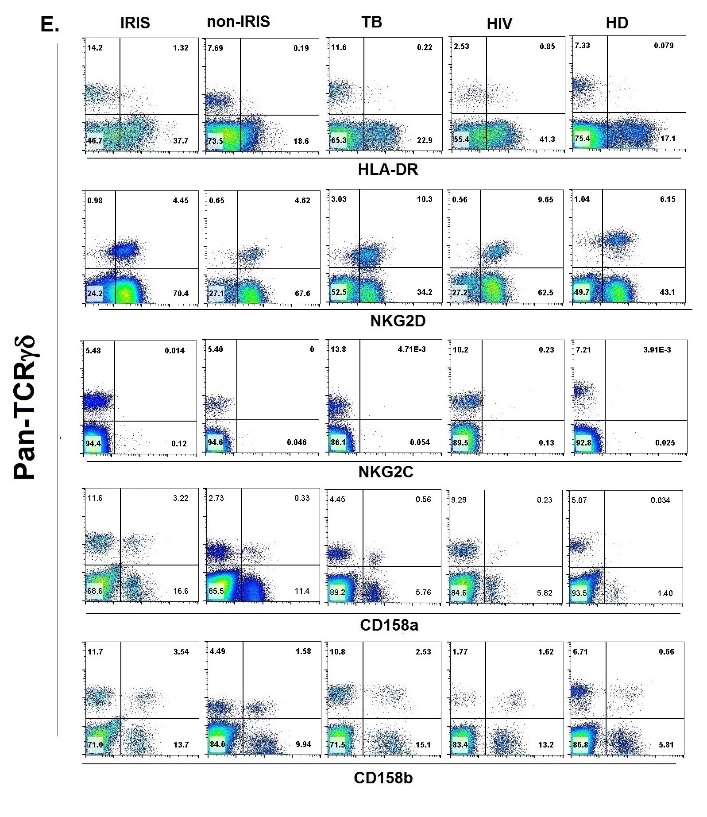
**

**Supplementary Figure 2 (Figure. S2).** **Flow cytometry analysis of different marker on gamma delta T cell, and on invariant NKT cells.** Representative flow cytometry plots of CD158a **(A.)**; CD158b **(B.)**; NKG2C **(C.)**; NKG2D **(D.)** expression on γδ^pos^ T cells; δ2^pos^γδ^pos^ T cells; δ2^neg^γδ^pos^ T cells**. (E.)** representative dot plot of HLA-DR; NKG2D; NKG2C; CD158a; CD158b versus pan-TCRγδ. Dot plots are analyzed in scatters gate of total lymphocytes.


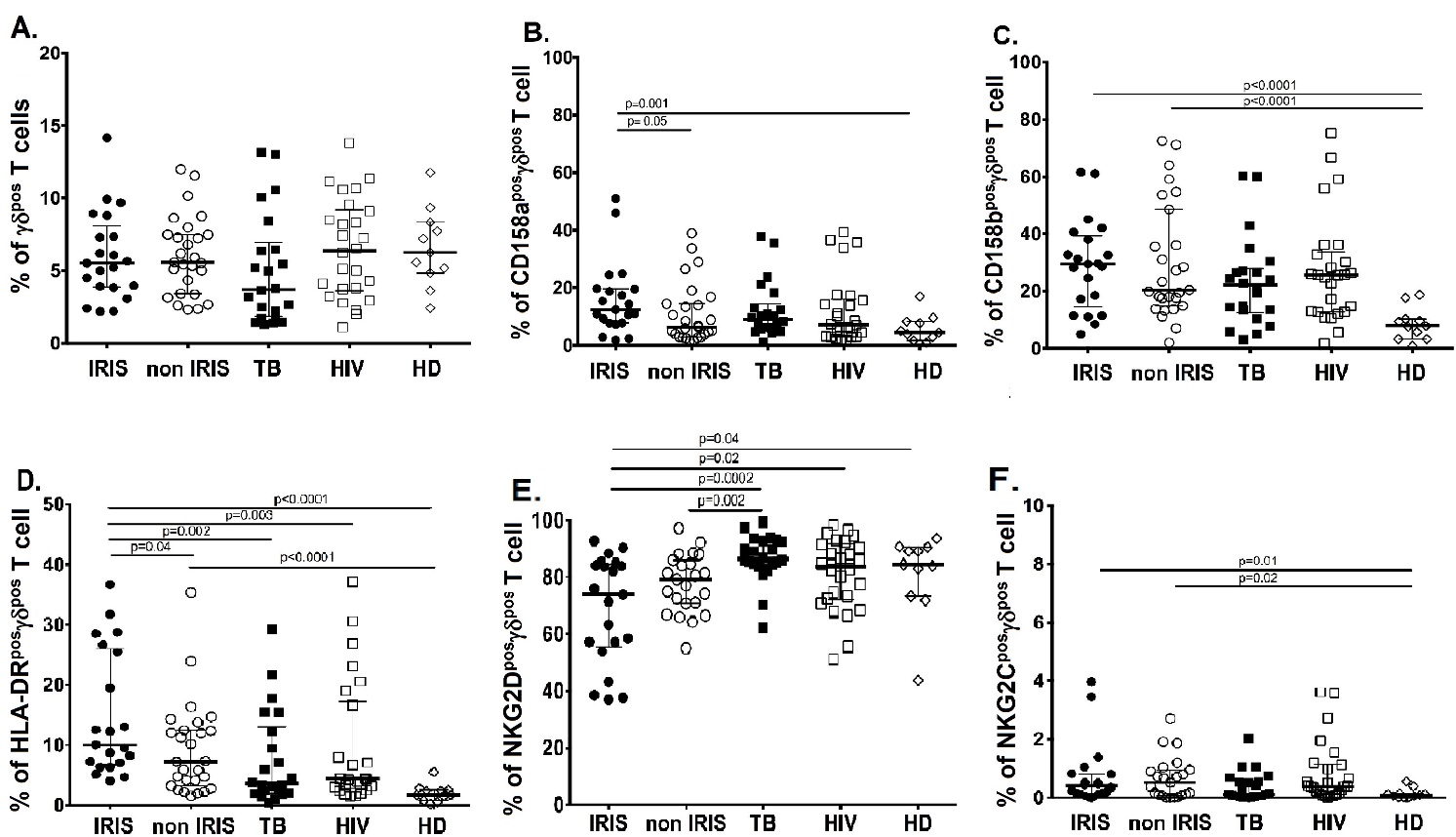


**Supplementary Figure 3 (Figure. S3).** **γδ^pos^ T cells and repertoire expression at the baseline in TB-IRIS and non-IRIS patients.** The proportion of γδ^pos^ T cells **(A.)**; CD158a^pos^γδ^pos^ T cells **(B.)**; CD158b^pos^γδ^pos^ T cells **(C.)**; HLA-DR^pos^γδ^pos^ T cells **(D.)**; NKG2D^pos^γδ^pos^ T cells **(E.)**; NKG2C^pos^γδ^pos^ T cells **(F.)** in TB-IRIS, non-IRIS, and control groups [TB (TB+/HIV-), HIV (HIV+/TB-) and HD (HIV-/TB-)] are shown. The results are expressed as median and 25% – 75% interquartile range of either γδ^pos^ T cells among lymphocytes **(A)** or among total γδ^pos^ T cells **(B – F)**. Significant p-values (p<0.05) are indicated.


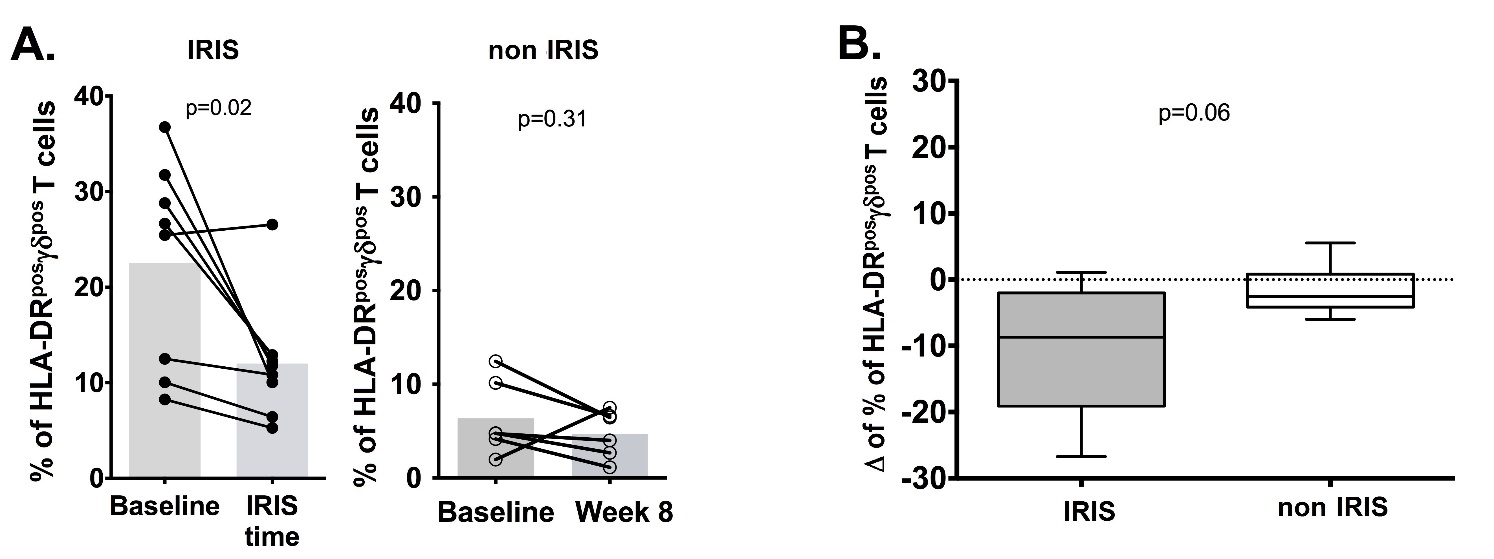


**Supplementary Figure 4 (Figure. S4).** **The evolution of HLA-DR expression on γδ^pos^ T cells at the time of IRIS in TB-IRIS and matched non-IRIS patients.**  The proportion of γδ^pos^ T cell expressing HLA-DR from baseline to IRIS time (W8 post-ART) **(A.);** and delta (Δ) values **(B.)** of TB-IRIS (n=8) and non-IRIS control (n=6) are shown. Results are expressed as median and 25% – 75% interquartile range. Significant p-values (p<0.05) are indicated.


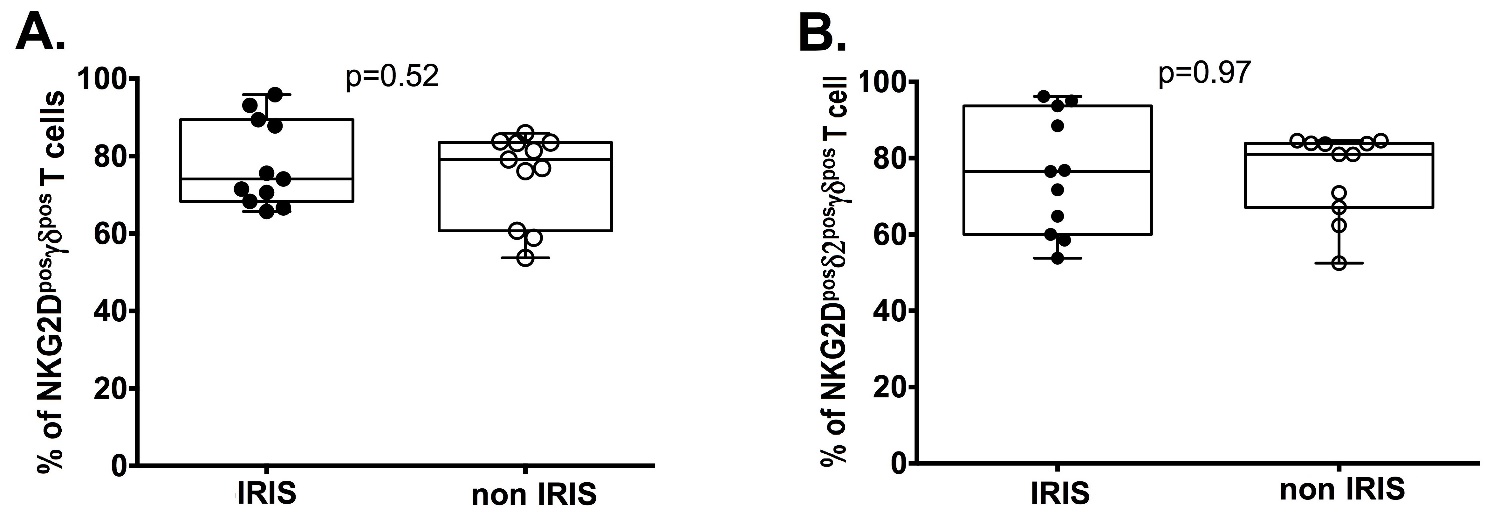


**Supplementary Figure 5 (Figure. S5). NKG2D expression on γδ^pos^ T cells, and δ2^pos^γδ^pos^ T cell subset at IRIS time.** The proportion of NKG2D^pos^γδ^pos^ T cells **(A.)**; NKG2D^pos^δ2**^pos^**γδ^pos^ T cells **(B.)** of TB-IRIS and non-IRIS control (matched IRIS time) are shown. The results are expressed as median and interquartile range. Significant p values (< 0.05) are indicated.


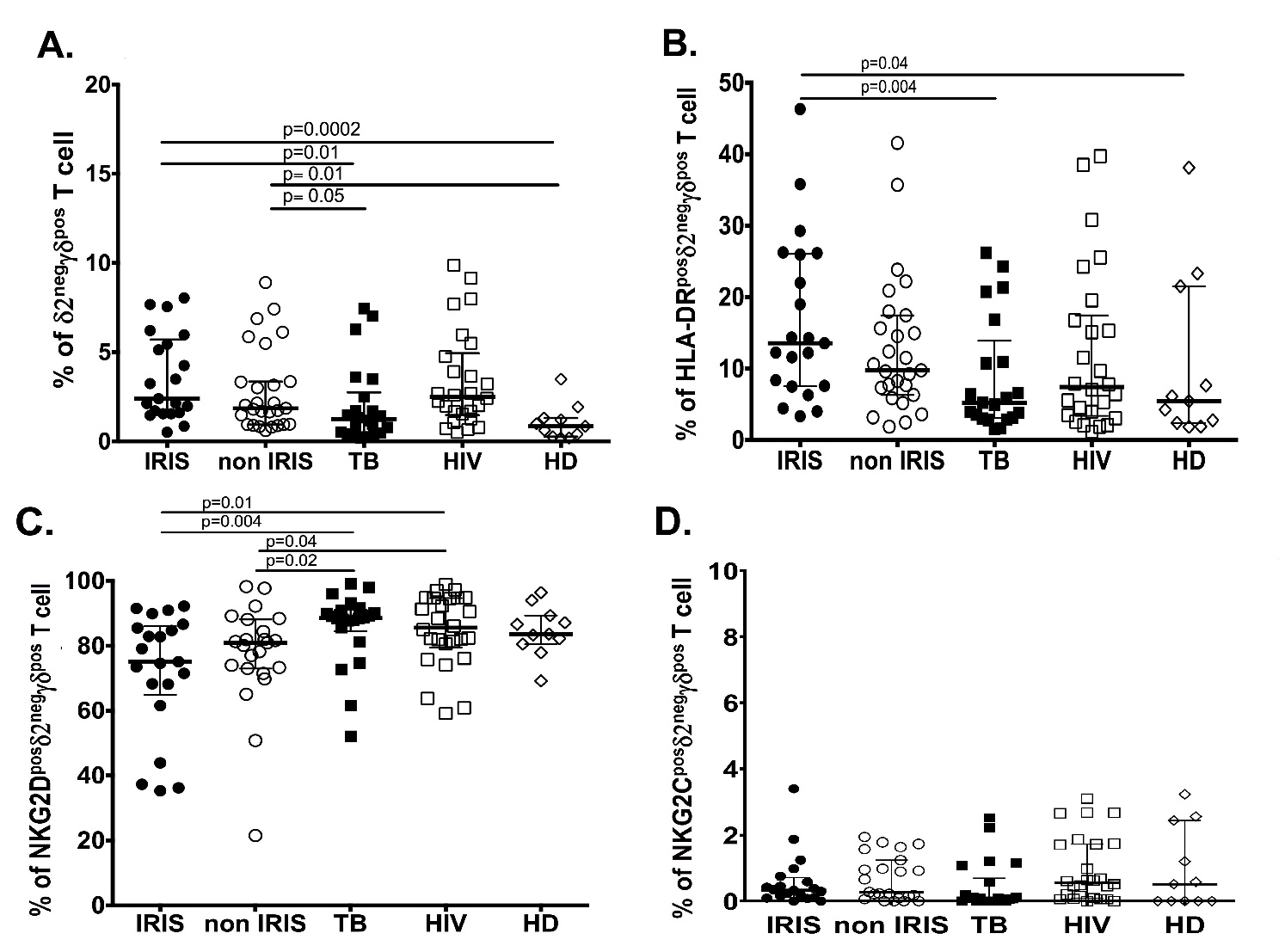


**Supplementary Figure 6 (Figure. S6).** **δ2^neg^γδ^pos^ subset and repertoire expression at baseline in TB-IRIS and non-IRIS.** The proportion of δ2^neg^γδ^pos^ T cells **(A.)**; HLA-DR^pos^δ2^neg^γδ^pos^ T cells **(B.)**; NKG2D^pos^ δ2^neg^γδ^pos^ T cells **(C.)**; NKG2C^pos^ δ2^neg^γδ^pos^ T cells **(D.)** in TB-IRIS, non-IRIS, and control groups [TB (TB+/HIV-), HIV(HIV+/TB-), HD (HIV-/TB-)] are shown. The results are expressed as median and 25% – 75% interquartile range. Significant p values (<0.05) are depicted.

**
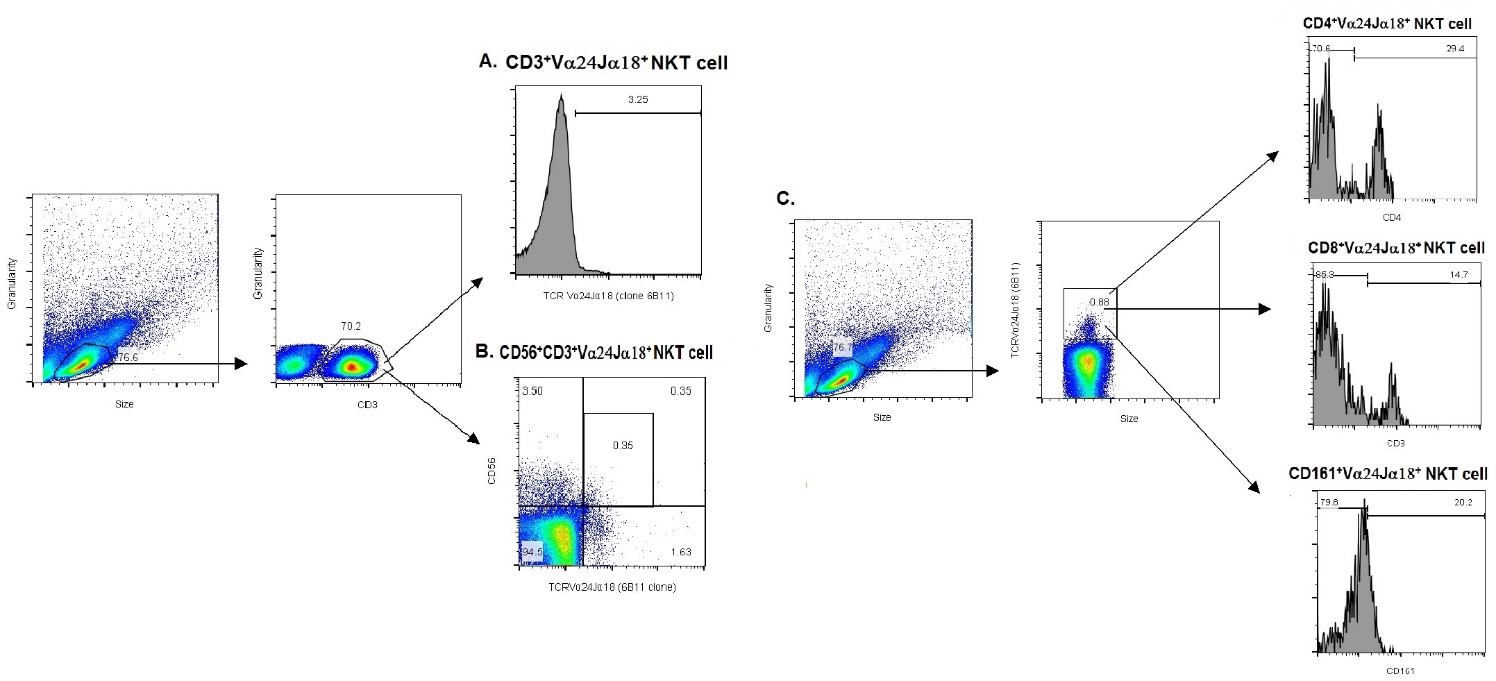
**

**Supplementary Figure 7 (Figure. S7).** **Gating strategy of invariant NKT cells.**
Flow cytometry plots of iNKT cells **(A.)**; CD56^+^iNKT cell **(B.)**; iNKT cell subset **(C.)** are shown. iNKT cells (CD3^+^Vα24Jα18 ^+^) and CD56^+^iNKT (CD56^+^CD3^+^Vα24Jα18^+^) are identified in CD3^+^ T cells. The expression of CD4; CD8; CD161 are analyzed in Vα24Jα18^+^. Invariant TCRVα24Jα18 was detected with 6B11 monoclonal antibody.

**
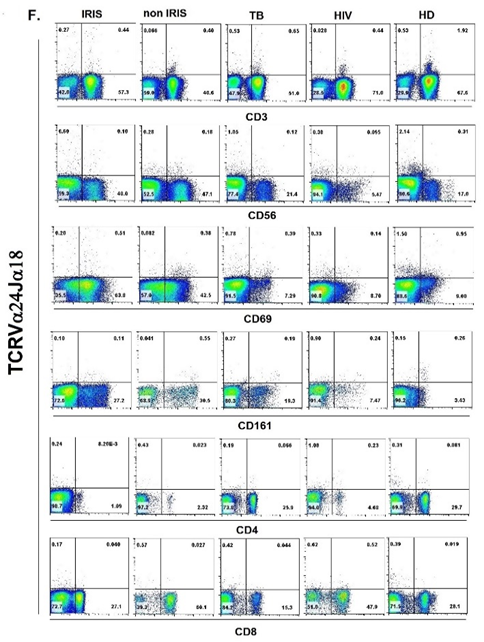

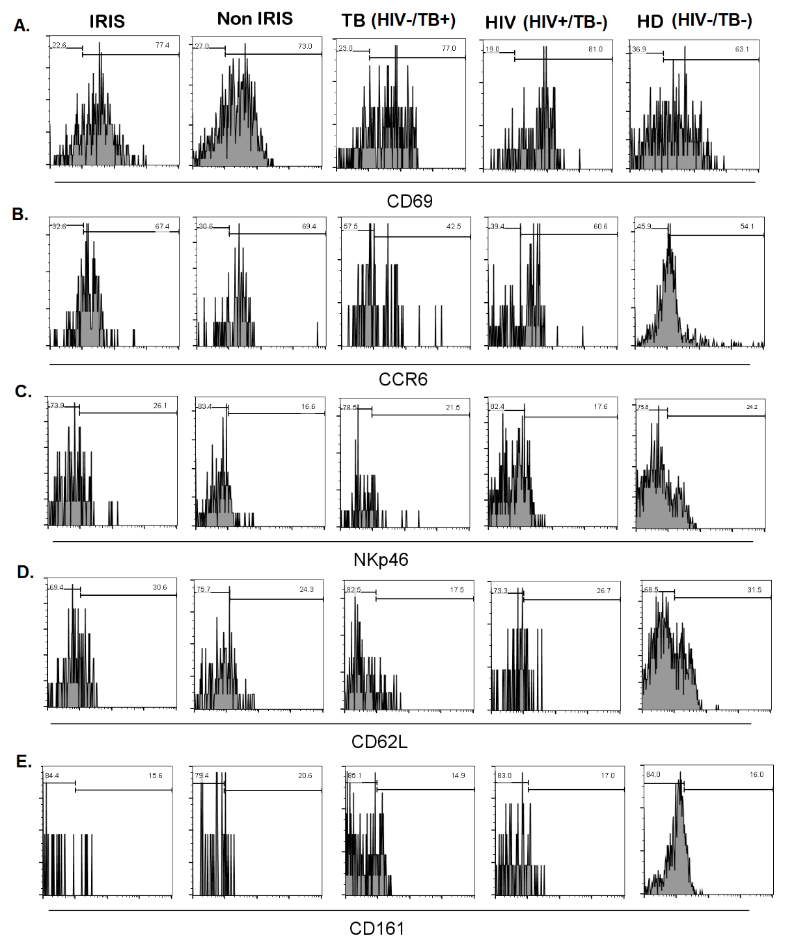
**

**Supplementary Figure 8 (Figure. S8). Flow cytometry analysis of the repertoire expression on CD56^+^ CD3^+^Vα24Jα18^+^ iNKT** **cells, and Vα24Jα18^+^ iNKT cells.** Representative flow cytometry histogram plots of CD69 **(A.)**; CCR6 **(B.)**; NKp46 **(C.)**; CD62L**(D.)**; CD161 **(E.)** expression among CD56^+^CD3^+^Vα24Jα18^+^ lymphocytes; and the dot plots of CD3; CD56; CD69; CD161; CD4; CD8 versus TCRVα24Jα18 **(F.)** among total lymphocytes are shown in different panels [IRIS, non-IRIS, TB (HIV-/TB+), HIV (HIV+/TB-) and HD (HIV-/TB-)].

**
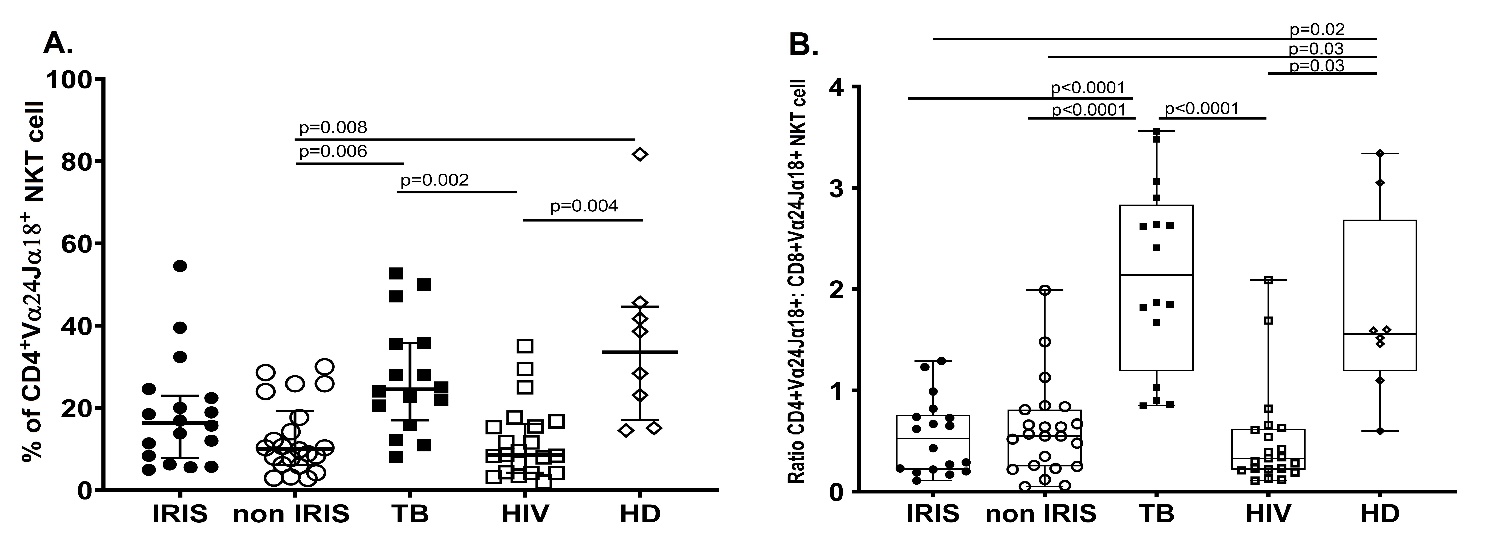
**

**Supplementary Figure 9 (Figure. S9). CD4^+^iNKT cells subset and the ratio of CD4^+^iNKT: CD8^+^iNKT cells**. CD4^+^ Vα24Jα18^+^ lymphocytes **(A);** ratio of CD4^+^ Vα24Jα18^+^: CD8^+^ Vα24Jα18^+^  **(B.)** of TB-IRIS, and non-IRIS at baseline, and control groups [TB (HIV-/TB+), HIV (HIV+/TB-), and HD (HIV-/TB-)] are represented. The results are expressed as median, 25%-75% interquartile range. Significant p values (p<0.05) are shown.

**Supplementary Table 1:** The combination of fluorochrome-conjugated monoclonal antibodies for immunostaining.

| Fluorochrome | FITC | PE | PC5 | APC |
| --- | --- | --- | --- | --- |
| **TCRγδ T cell** | | | | |
| Panel 1 | TCR Vδ2 | CD158a | Pan-TCRγδ | HLA DR |
| Panel 2 | TCR Vδ2 | CD158b | Pan-TCRγδ | HLA DR |
| Panel 3 | TCR Vδ2 | NKG2D | Pan-TCRγδ | NKG2C |
| **invariant NKT cell** | | | | |
| Panel 4 | CD3 | CD69 | CD56 | TCRVα24Jα18 (6B11) |
| Panel 5 | CD3 | NKp46 | CD56 | TCRVα24Jα18 (6B11) |
| Panel 6 | CD3 | CD62L | CD56 | TCRVα24Jα18 (6B11) |
| Panel 7 | CD3 | CCR6 | CD56 | TCRVα24Jα18 (6B11) |
| Panel 8 | CD4 | CD161 | CD56 | TCRVα24Jα18 (6B11) |
| Panel 9 | CD8 | CD161 | CD56 | TCRVα24Jα18 (6B11) |
